# From words to pixels: The Infectious Diseases in Motion (IDIM) and VACCELERATE experience for fast and accessible science audiovisual communication

**DOI:** 10.1016/j.onehlt.2023.100648

**Published:** 2023-11-07

**Authors:** Jon Salmanton-García, Janina Leckler, Oliver A. Cornely

**Affiliations:** a Faculty of Medicine and University Hospital Cologne, Institute of Translational Research, Cologne Excellence Cluster on Cellular Stress Responses in Aging-Associated Diseases, University of Cologne, Cologne, Germany; b Department I of Internal Medicine, Faculty of Medicine and University Hospital Cologne, Excellence Center for Medical Mycology, University of Cologne, Cologne, Germany; c German Centre for Infection Research (DZIF), Partner Site Bonn-Cologne, Cologne, Germany; d University of Cologne, Faculty of Medicine and University Hospital Cologne, Clinical Trials Centre Cologne (ZKS Köln), Cologne, Germany

**Keywords:** Internet impact, Accessible science information, COVID-19, Digital scientific outreach, Infectious diseases

## Abstract

**Background:**

The internet's impact on knowledge distribution has led to a growing demand for accessible science information. COVID-19 heightened interest in science, emphasizing the need to combat misinformation. This publication discusses digital scientific outreach, particularly in infectious diseases, to counter misinformation and promote evidence-based communication.

**Methods:**

Infectious Diseases in Motion (IDIM) and VACCELERATE use YouTube for infectious diseases and vaccine research dissemination. They create video abstracts by identifying relevant publications, coordinating recording sessions, and producing visually engaging content.

**Results:**

As of August 2023, IDIM and VACCELERATE have produced 173 videos, attracting viewers from 34 countries. This global reach supports their role as valuable resources for the international scientific community.

**Conclusions:**

The success of these initiatives lies in inclusivity, collaboration, multilingual content, and effective promotion. They aim to become essential hubs for global scientific knowledge dissemination, advancing understanding through shared knowledge.

## Highlights

• IDIM and VACCELERATE YouTube channels stress accessible science's key role in countering misinformation.
• IDIM and VACCELERATE use YouTube with 173 videos by August 2023, showcasing it as a platform for sharing scientific knowledge.
• IDIM and VACCELERATE engage viewers from 34 countries through multilingual content, emphasizing their global reach.
• A science YouTube channel elevates a research group's global reach and promotions.

## 1 Introduction

The way knowledge is distributed has changed significantly as a result of the internet and the rise of virtual platforms. More people from all backgrounds are turning to online resources [1], especially for information, including science-related topics [2]. The need for easily comprehensible and accessible scientific information grows along with scientific research and advancements. [3] In recent years, the appeal of audiobooks [4,5], audio papers [6], and other audio-visual media [7] has skyrocketed. This trend might be driven by people's limited ability to read scientific literature in-depth [7]. As a result, in order to respond to this changing environment and increase the accessibility of research findings, many scientific publications now encourage or even demand the use of alternative data presentation techniques, such as graphical abstracts and video abstracts [8], has been reported to be associated with increased dissemination.

The coronavirus disease 2019 (COVID-19) pandemic has also significantly impacted how society views science [9]. As people became more conscious of the importance of scientific advancements and study, they started incorporating scientific elements into their regular conversations. This interest has been sparked by discussions on vaccination research, public health policy, and preventative measures [10]. However, it is important to note that this phenomenon extends beyond infectious diseases [11], as people are becoming more eager to investigate scientific problems across a range of fields [12]. Moreover, fighting against fake news in science is crucial to prevent the spread of misinformation and inaccuracies that can lead to public confusion and distrust in scientific findings. This misinformation can have serious consequences on public health, policy-making, and the advancement of knowledge. Scientists, researchers, journalists, and educators must promote transparency, evidence-based reporting, and fact-checking to uphold the integrity of scientific information [13]. By countering fake news in science, we can empower individuals to make informed decisions and support the progress of science for the benefit of all [14,15].

The goal of this publication is to quickly, and efficiently convey our knowledge and experiences in digital scientific outreach. This strategy tries to make our work more accessible to those with hectic schedules by reducing the need for prolonged reading. Furthermore, we focus on the responsibility of countering misinformation about infectious diseases and associated topics. Eventually, we aim to encourage informed dialogue and supply both specialists and the general public with trustworthy scientific knowledge by presenting correct research.

## 2 Overview

Infectious Diseases in Motion (IDIM, https://youtube.com/@idiminfectiousdiseasesinmo6269) is a pioneering initiative focused on infectious diseases research dissemination, leveraging the power of YouTube to spread scientific knowledge rapidly. The main objective of IDIM is to provide video abstracts that summarize the latest scientific findings and hot topics in the field of infectious diseases. These video abstracts are designed to cater to individuals with limited time, allowing them to gain insights into ongoing research activities with just a glimpse. By presenting information in a visually captivating and easily digestible manner, IDIM bridges the gap between researchers and the broader public, enabling greater awareness and understanding of infectious diseases. In parallel with IDIM, VACCELERATE [16,17] (www.vaccelerate.eu, https://youtube.com/@vaccelerate_eu919) is an initiative with a specific focus on vaccine research and development. The primary goal of VACCELERATE is to develop vaccine phase 2 and 3 trials, promoting in the meantime vaccine awareness by different means, including video abstracts and online seminars.

**Fig. 1:**
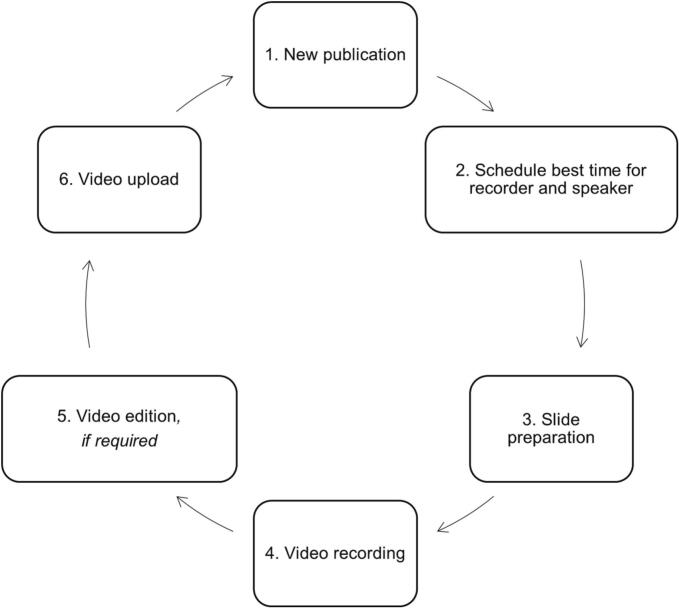
Process of creating video abstracts in IDIM and VACCELERATE YouTube channels.

As shown in Fig. 1, the first pivotal step involves meticulously identifying new and relevant publications in infectious diseases, ensuring that the video abstracts stay abreast of the latest developments and research findings in the field. This enables the platform to provide cutting-edge and up-to-date information to its audience. Another critical aspect of the process is the careful coordination of recording sessions and speaker availability, optimizing the utilization of time and resources. This ensures that the video abstracts are delivered with the utmost clarity and expertise, as experts in the respective fields present the information concisely and comprehensively. Equally important is the slide preparation phase, where the information is thoughtfully curated and visually represented to enhance understanding and engagement. The design and layout of the slides are carefully considered to create an impactful and memorable experience for the audience. The recording process itself is conducted with great attention to detail, with an emphasis on clarity, quality, and professionalism. Each video abstract is crafted to present the key insights and findings in a succinct and captivating manner, making it accessible to a wide range of audiences, from experts to enthusiasts. Timely uploads are a critical aspect of the initiative, as they ensure that the information reaches the audience promptly.

**Fig. 2:**
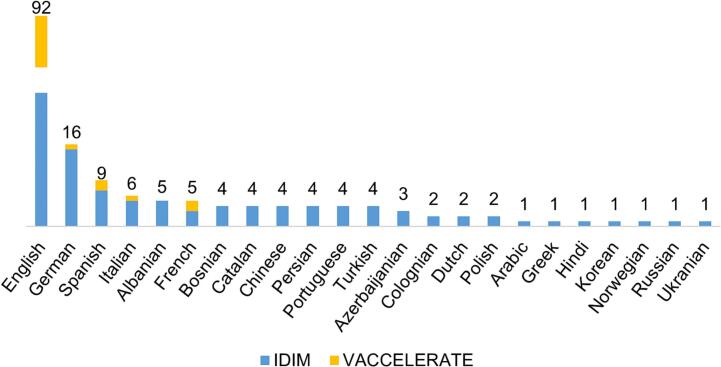
Number of videos in IDIM and VACCELERATE YouTube channels per language. **IDIM**, Infectious Diseases in Motion.

As of August 2023, an extensive collection of 173 videos between both channels has been generated (IDIM 127 videos, VACCELERATE 46 videos), catering to diverse interests and providing valuable insights into the latest advancements and discoveries in infections, such as invasive fungal infections, viral infections, and vaccines. One of the platform's most notable features is its commitment to inclusivity, exemplified by the provision of content in multiple languages (Fig. 2) and different target audiences: children, adults, or researchers. By breaking language and audience barriers, the platform seeks to connect with a global audience, promoting cross-cultural understanding and collaboration among researchers and science enthusiasts worldwide.

**Fig. 3:**
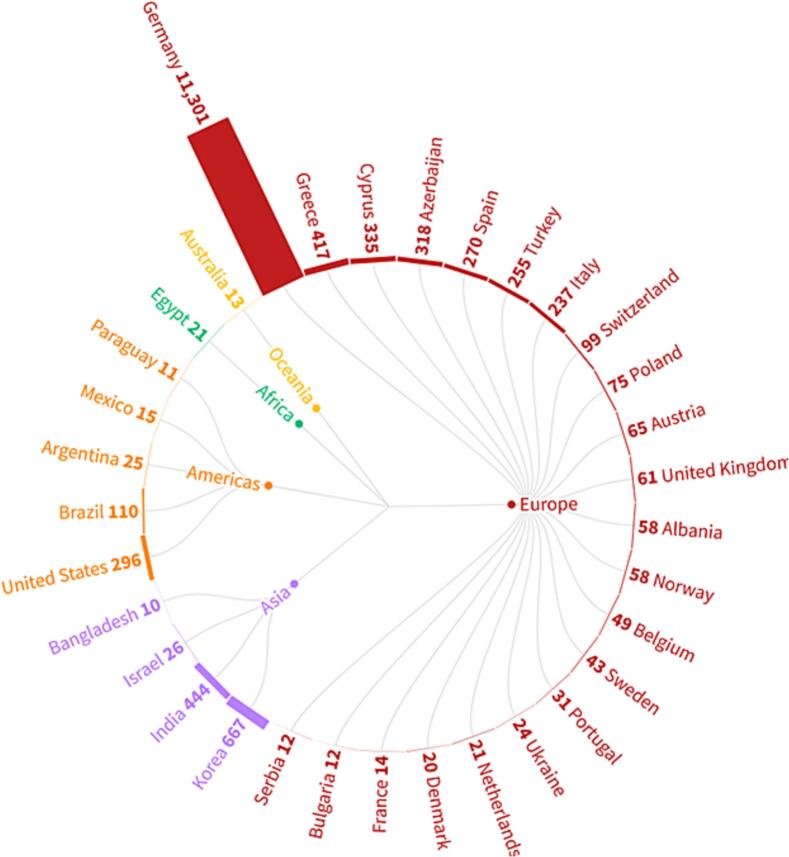
Number of video views in IDIM and VACCELERATE YouTube channels per country.

As a testament to its success, the platform has garnered visits from 34 countries (33 IDIM, 8 VACCELERATE, partly overlapping) (Fig. 3). This global outreach underscores the platform's significance as a valuable resource for the international scientific community and further fuels its mission to disseminate knowledge and foster a culture of evidence-based discussions and decision-making.

## 3 Outlook

The platform's approach extends a warm and enthusiastic invitation to external participants, including esteemed experts and researchers from relevant publications in infectious diseases. Embracing diversity and collaboration, the platform seeks to create a vibrant and dynamic community, where individuals with different perspectives and backgrounds come together to share their insights and expertise. By actively engaging these external participants, the platform aims to nurture a culture of knowledge exchange and open dialogue, driving forward the boundaries of scientific understanding.

Acknowledging the global nature of scientific inquiry, the platform recognizes the importance of breaking language barriers [18,19]. To achieve this, it takes significant strides in ensuring that its valuable video content is made available in further languages, making science more accessible to a wider audience worldwide. This multilingual approach not only reaches individuals who may have previously faced language constraints but also promotes cross-cultural exchange, fostering a richer and more interconnected global scientific community.

As an ever-evolving repository of knowledge, the platform is dedicated to staying at the forefront of scientific advancements. It proactively explores and incorporates further topics that are of interest and relevance to the community it serves. This commitment to providing up-to-date and comprehensive information empowers users with the latest research findings, emerging trends, and breakthroughs across a myriad of disciplines, enriching their understanding and expanding the horizons of scientific exploration.

Recognizing that the dissemination of knowledge relies on effective outreach [1], the platform embraces a multifaceted approach to promotion. Leveraging various channels and strategies, it seeks to attract and engage new subscribers, sharing the platform's unique offerings and benefits. The team behind the platform continuously analyses and refines its promotional efforts, ensuring that they resonate with the intended audience and yield meaningful results. This dedication to improvement and adaptation enables the platform to maintain sustained growth in its user base and secure its position as a trusted and invaluable resource in the scientific community.

In essence, the platform's overarching goal is to become a pivotal hub for scientific knowledge dissemination, collaboration, and community-building on a global scale. By fostering an inclusive and collaborative environment, breaking language barriers, remaining at the forefront of research, and employing strategic promotion, the platform aspires to play a transformative role in advancing scientific understanding and positively impacting the world through the power of shared knowledge.

## Funding

Not applicable.

## Contribution statement

JSG, JL, and OAC conceived the study idea. JSG wrote the initial manuscript draft. JSG, JL, and OAC revised and approved the manuscript.

## Ethics approval

Not applicable.

## Declaration of Competing Interest

Authors declare no conflict of interest regarding the current work.

## Data Availability

Data can be made available after a reasonable requestion following the paths described in the manuscript.
